# Moving Beyond Technique: A Conceptual Perspective for the Cuevas Medek Exercise Aligned with Modern Neuroscience

**DOI:** 10.3390/children13060769

**Published:** 2026-06-01

**Authors:** Cristiano Becker, Juliana Barbosa Goulardins, Juliana Cristina Fernandes Bilhar Marques, Ramon Jeronimo Cuevas Gajardo

**Affiliations:** 1 CB Center Therapies and CME Courses, Cunha Porã 89890-000, SC, Brazil; contato@centrocb.com.br; 2 Escola Bahiana de Medicina e Saúde Pública, Salvador 40290-000, BA, Brazil; 3 Neurologic Rehabilitation Clinic, São Paulo 03410-010, SP, Brazil; 4 CME Therapy Spa, Santiago 7550000, Chile

**Keywords:** pediatric rehabilitation, motor skills, neuroplasticity, Cuevas Medek Exercise, physical therapy

## Abstract

**Highlights:**

**What are the main findings?**

- Cuevas Medek Exercise can be conceptually aligned with contemporary frameworks of motor control, developmental neuroscience, and neuroplasticity.
- Its core principles can be understood as structured sensorimotor opportunities that may support adaptive motor development.

**What are the implications of the main findings?**

- This conceptual reframing may support clearer clinical reasoning and provide a theoretical basis for future investigation of CME within contemporary pediatric rehabilitation frameworks.
- It provides a theoretical foundation to guide future research on mechanisms, intervention fidelity, and clinical effectiveness.

**Abstract:**

Despite its increasing use in pediatric neurorehabilitation, Cuevas Medek Exercise (CME) remains rooted in clinical tradition, with limited theoretical articulation in relation to contemporary models of motor development, control, and learning. While clinical observations and reports from practice suggest possible functional benefits in children with motor delays, the absence of a conceptual framework limits its integration into evidence-informed physical therapy practice and education. This perspective proposes a conceptual model for CME that aligns its core principles with current theoretical constructs in motor behavior and developmental neuroscience. By examining key elements of CME—such as distal initiation, postural challenge, and task variability—through the lens of affordances, self-organization, and experience-dependent plasticity, the article presents CME not merely as a technique, but as a conceptual developmental approach informed by embodied action. We argue that such reframing may support more rigorous clinical reasoning, contribute to interdisciplinary dialogue, and inform a theoretical basis for future research exploring the proposed mechanisms and potential effects of CME. More broadly, this perspective contributes to ongoing discussions on how clinically derived interventions can be conceptually integrated within contemporary rehabilitation science. While the present article does not provide empirical validation, it offers a theoretical framework intended to inform future investigation and critical reflection in pediatric physical therapy.

## 1. Introduction

Pediatric neurorehabilitation is increasingly shaped by advances in neuroscience, motor control, and developmental theory [1,2]. Therapy interventions have evolved in line with the World Health Organization’s International Classification of Functioning, Disability and Health (ICF) framework, shifting toward activity-based and meaningful tasks [3]. This shift requires clearer conceptual articulation of therapeutic approaches, enabling transparency, replicability, and scientific rigor [4,5].

Among approaches in pediatric therapy, Cuevas Medek Exercise (CME) has gained attention for its distinctive emphasis on distal input, active motor responses, and progressive exposure to gravity as therapeutic strategies intended to encourage postural control and functional motor learning [6,7,8]. Despite its growing clinical use, CME faces challenges in aligning its conceptual structure with contemporary theoretical models.

Contemporary frameworks, including experience-dependent neuroplasticity [9,10], sensorimotor integration [11], and affordance-based approaches [12,13], provide a basis to reinterpret therapeutic methods as dynamic systems of embodied learning. Aligning traditional therapeutic methods with contemporary theories through a shared conceptual language may support integration between research and practice.

The purpose of this Perspective is to propose a conceptual framework for CME by aligning its core therapeutic components with contemporary constructs from developmental neuroscience, motor control, and pediatric rehabilitation. This article is intended as a conceptual perspective rather than a systematic review and does not aim to provide empirical validation of CME. Instead, it aims to support clinical reasoning and generate theoretical hypotheses for future research.

## 2. Basic Principles

To support a conceptual analysis of CME, it is necessary to understand the therapeutic principles that guide its application. Developed by Ramón Cuevas in the 1970s, CME emerged from clinical practice with children with cerebral palsy and other motor delays, based on an intervention logic centered on the early activation of the postural system through intensive and challenging stimuli [6]. The main principles that guide its clinical practice are described below, which will be discussed in light of contemporary theories in neuroscience, motor control, and child development.

**Figure 1:**
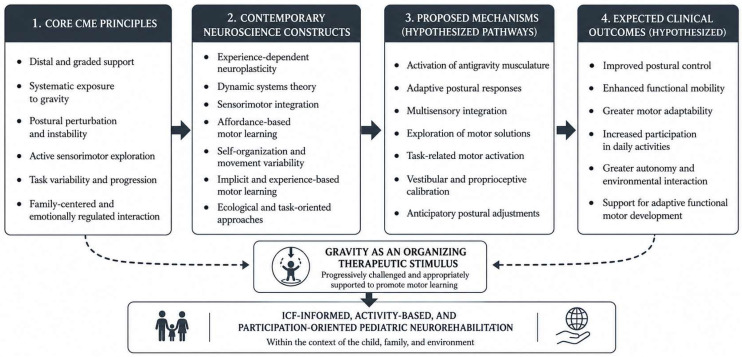
Conceptual framework of CME aligned with contemporary developmental neuroscience and motor behavior theories. The figure summarizes the proposed relationships between core CME therapeutic principles, contemporary neuroscience constructs, hypothesized neurobehavioral mechanisms, and expected clinical outcomes. The model is conceptual and intended to support clinical reasoning and future hypothesis-driven research, rather than represent empirically validated causal pathways. Arrows represent proposed conceptual relationships and hypothesized interactions between domains within the framework.

Figure 1 summarizes the proposed relationships between CME therapeutic principles, contemporary neuroscience constructs, hypothesized mechanisms, and potential clinical implications.

### 2.1. Stimulation of the Emergence of Absent Motor Functions

CME is grounded in the premise that, even in the presence of significant motor deficits, the developing nervous system retains latent potential for the emergence of new motor functions. The central aim of the approach is to encourage previously absent functional motor responses—such as postural control in unsupported standing—through specific therapeutic manipulations that directly challenge the child’s existing motor repertoire [6]. This principle is conceptually aligned with several contemporary theoretical frameworks that recognize the capacity of the nervous system to undergo experience-driven reorganization during development.

Experience-dependent neuroplasticity provides a well-established foundation for this approach. Neural circuits can be reshaped through repetitive, functionally meaningful sensorimotor stimulation, even in the context of early brain injury or immaturity [14,15]. In parallel, dynamic systems theory posits that motor behavior emerges from the interaction of multiple systems (neural, biomechanical, environmental), and that small changes in environmental or bodily parameters—such as those introduced during CME—may contribute to the emergence of new motor patterns [16,17]. From an epigenetic perspective, motor development results from self-organizing processes that are sensitive to environmental inputs, emphasizing the crucial role of early movement experiences in shaping neural circuitry [18]. Additionally, task-oriented motor learning models underscore the importance of structured, goal-directed challenges for skill acquisition, even when voluntary control is limited. These models highlight variability, task specificity, and repetition as key elements associated with motor learning and functional adaptation [19,20].

Despite occasional critiques framing CME as a passive intervention [21,22]—particularly due to its reliance on therapist-applied movements—this characterization may not fully reflect the therapeutic rationale and sensorimotor assumptions underlying the approach. These critiques also reflect broader ongoing discussions in pediatric neurorehabilitation regarding the role of therapist-driven facilitation approaches in comparison with task-specific, child-initiated, and participation-oriented intervention models. Contemporary rehabilitation frameworks increasingly emphasize active engagement, environmental interaction, and goal-directed practice as central elements of motor learning. Within this context, approaches involving manual facilitation or therapist-guided handling continue to generate debate regarding the balance between external guidance and self-initiated motor exploration.

Unlike passive range-of-motion exercises, CME does not aim to move the child’s body in place of their own motor system; rather, it seeks to encourage active postural reactions and neuromuscular responses by exposing the child to strategically induced gravitational challenges and perturbations. These perturbations are intended to promote sensorimotor adaptation by encouraging reflexive and anticipatory motor responses that may emerge independently of conscious volition [23,24]. In this sense, CME aligns with contemporary views on embodied action and implicit motor learning, which recognize that motor skills can be acquired not only through deliberate repetition but also through task-relevant sensorimotor challenges that may engage intrinsic neural resources [25,26]. When appropriately understood, CME represents a bottom-up, sensorimotor-based strategy that is proposed to engage the child’s residual adaptive capacity to organize motor output—particularly in populations with limited volitional control—thus framing CME as an approach intended to encourage active postural and sensorimotor engagement rather than passive movement execution.

### 2.2. Promotion of Automatic Postural Responses

Another core principle of CME is the facilitation of automatic postural responses, without requiring conscious collaboration or voluntary motor initiation from the child. Rather than relying on a hierarchical model of motor control, this principle reflects a contemporary understanding of postural behavior as an emergent property of multiple interacting systems—including sensory, motor, biomechanical, and environmental subsystems. In this view, postural adaptations are not “triggered” by brainstem or subcortical centers in isolation but arise from the interplay between intrinsic organismic constraints and extrinsic task demands, particularly under gravitational influence [16,17,24].

CME therapeutic maneuvers and activities are intentionally designed to manipulate these task and environmental variables—such as changes in support surface, alignment, or load distribution—in order to encourage adaptive postural adjustments. These are understood not as mere reflexes, but as adaptive motor solutions that emerge in response to task perturbations [27,28]. Within this framework, CME draws on the non-linear and distributed nature of motor behavior to encourage adaptive postural responses through sensorimotor experience. The variability elicited through these perturbation-based strategies may encourage exploration, calibration, and refinement of postural strategies, aligning with current views of adaptive motor development [17].

In CME, verticalization refers to a continuum of graded postural challenges—ranging from prone and rolling activities to sitting, quadruped, kneeling, standing, and gait. Each posture is selected and progressed based on a functional assessment of the child’s current abilities and therapeutic goals, aiming to challenge specific components of head control, trunk stability, antigravity activation, or functional mobility. The therapist calibrates postural and gravitational demands to the child’s capacity, with the intention of maintaining each experience achievable while sufficiently challenging to promote learning.

Thus, CME incorporates both proximal and distal handling strategies across diverse positions, not as ends themselves but as vehicles for supporting interaction with gravity and the environment. This graded approach reflects an ecological and developmental rationale in which interaction with gravity and task demands may contribute to postural adaptation and motor coordination [29,30]. By embedding these challenges into functional contexts—such as transitions, reaching, crawling, or supported walking—CME aims to encourage automatic postural responses that are directly relevant to everyday movement and participation.

### 2.3. Systematic Exposure to Gravity as a Therapeutic Stimulus

In CME, gravity is approached as a central therapeutic component. The method strategically exposes specific body segments to gravitational demands in a progressive and calibrated manner, using biomechanical challenges intended to encourage antigravity muscle activation and postural adaptation. This principle aligns with task-oriented postural control theories, which emphasize that functional postural strategies emerge through interaction between intrinsic biomechanical constraints and environmental forces—chief among them, gravity [19,31].

From a biomechanical and developmental perspective, sustained gravitational loading is considered an important factor in shaping musculoskeletal structure and function. In typical development, early vertical orientation and weight-bearing experiences contribute to the maturation of spinal curves, axial alignment, and the activation of deep postural muscles [32,33]. In children with delayed or limited mobility, this exposure is often lacking. CME addresses this gap by systematically introducing antigravity demands that may encourage axial extension, weight shifting, and antigravity postural responses. This rationale is theoretically consistent with dynamic systems theory, which proposes that gravity may function as a “control parameter”—a variable that, when manipulated, may influence self-organization and adaptive motor responses [16,17]. Through carefully structured perturbations, CME proposes controlled postural challenges intended to encourage exploration of adaptive strategies for upright posture.

Notably, clinical studies in populations at risk for motor impairment—such as preterm infants and children with cerebral palsy—highlight the consequences of insufficient or delayed exposure to gravitational challenges during early development. In preterm infants, the lack of consistent upright experience and antigravity postural challenge has been associated with delayed maturation of spinal curves, hypotonia, and poor axial alignment, often resulting in compensatory strategies that limit functional mobility [34,35]. Similarly, children with spastic cerebral palsy often exhibit underdeveloped extensor muscle activity and poor postural orientation due, in part, to limited early engagement with vertical support and gravitational demand [36,37,38].

These clinical and neurophysiological patterns may support the theoretical relevance of graded gravitational loading, as proposed in CME. Within this framework, gravitational exposure is approached as a carefully graded process that considers the child’s specific challenges and functional capacity. Rather than passive or unstructured exposure to gravity, CME proposes calibrated postural and sensorimotor challenges intended to encourage active postural engagement within functional environments in developmentally meaningful contexts.

### 2.4. Sensorimotor Stimulation Through Graded Support

A defining feature of CME is its dynamic handling strategy, which progressively transitions from proximal to distal support according to the child’s individual level of postural control and functional capacity. The therapist’s choice of support is guided by continuous assessment of the child’s postural control, alignment, and adaptive responses.

When clinically appropriate, distal support is introduced to provide a controlled challenge to the neuromotor system. By reducing external stabilization near the body’s center of mass, the therapist may encourage increased axial muscle engagement and sensorimotor processes involved in postural orientation and balance [19,39]. From a biomechanical perspective, manipulating the point of contact modifies the direction and magnitude of force vectors acting on the body, increasing the degrees of freedom involved in maintaining stability [28,40].

This graded handling approach aligns with contemporary theories of motor learning and neuroplasticity, emphasizing that varied motor experiences may contribute to proprioceptive and vestibular processing during development [9]. CME’s rationale also draws from constraint-based theories of motor learning, which propose that manipulating tasks and environmental constraints may encourage self-organization [16,41]. Within this framework, distal support functions as a therapeutic constraint that, when appropriately timed and dosed, may encourage the child to explore new postural strategies and intersegmental coordination patterns.

Importantly, CME does not approach distal handling as a fixed rule, but rather as a therapeutic progression intended to encourage autonomy, movement variability, and active engagement. Through structured exposure to graded challenges, the child is provided with opportunities for sensorimotor exploration and adaptive responses within functionally relevant contexts, positioning therapist handling as an active therapeutic component rather than a passive maneuver.

### 2.5. Functional Mobility and Dynamic Stretching Integrated into Task Execution

In CME, mobility and flexibility are addressed not through isolated stretching techniques, but via dynamic mobilization integrated into functional, weight-bearing activities. This approach is consistent with high-level evidence suggesting that passive stretching alone offers minimal clinical benefit, especially in pediatric neurorehabilitation. The State of the Evidence Traffic Lights 2019 review categorized passive stretching as a “red light” intervention for children with cerebral palsy—indicating that it lacks efficacy and may incur opportunity costs when used in place of more functional strategies [1]. Recently, an umbrella systematic review of postural interventions concluded that task-specific and posture-integrated approaches were associated with more favorable outcomes, while non-task-based methods, including passive techniques, were less likely to be associated with meaningful outcomes [21].

From a physiological standpoint, systematic reviews in healthy populations have shown that acute static stretching can transiently reduce muscle performance, with limited effects on long-term range of motion unless combined with active movement or strength training [21,42]. CME attempts to address these limitations by embedding joint mobilization and soft tissue extensibility within sensorimotor activities that simultaneously engage postural control, alignment, and neuromuscular activation. This strategy may support functional range of motion while remaining conceptually aligned with principles of motor learning and neuroplasticity that emphasize task relevance, active participation, and integration of multiple motor subsystems.

### 2.6. Emotional Regulation and Family-Centered Practice in Therapeutic Interaction

While often overlooked in biomechanical frameworks, the child’s emotional state may influence engagement in sensorimotor interventions. CME recognizes that many children with motor impairments exhibit heightened emotional reactivity and reduced tolerance to novelty and physical challenge, due in part to altered sensory processing and limited opportunities for exploratory movement. As such, the method emphasizes a therapeutic posture marked by calm affect, firm but gentle handling, and empathic attunement to the child’s responses. These strategies align with contemporary models of co-regulation, in which the caregiver or therapist supports the child’s autonomic and emotional regulation [43,44].

From a neurodevelopmental perspective, emotional security may play an important role in motor learning by supporting attentional engagement, sensorimotor exploration, and the integration of novel experiences [45]. When children feel unsafe, dysregulated states may be associated with exaggerated primitive reflexes or avoidance behaviors, inhibiting the emergence of adaptive postural strategies—an observation consistently reported in CME clinical practice.

Crucially, CME adopts a family-centred approach, recognizing that therapeutic engagement may depend not only on in-session strategies but also on collaboration with families, caregivers, and peers. The therapist-family partnership is considered central to the method. Through shared decision-making, caregivers are guided to understand the therapeutic goals, observe the child’s cues, and reinforce daily opportunities for active movement and postural engagement. The Home Program—tailored to the child’s abilities and family context—may support continuity of therapeutic activities beyond the clinical setting and encourage consistency and generalization within the child’s natural environments. Family participation in following the therapist’s guidance and maintaining therapeutic routines is viewed not as ancillary, but as an important component of the intervention process [46,47].

Recent neurorehabilitation frameworks further emphasize that motivation and task salience are not ancillary, but meaningful contributors to neuroplastic processes [48]. These principles converge with the F-words for Child Development—especially the dimension of “fun”—which frames joyful and engaging participation as central to participation and functional development [49]. In CME, therapeutic interactions are therefore designed not only to challenge the child physically but also to support emotional and motivational attunement within a safe and collaborative environment.

### 2.7. Early Vertical Stimulation in the Presence of Tone Alterations

CME offers an alternative perspective regarding traditional hesitations around introducing upright positioning in infants presenting with tone abnormalities, particularly lower-limb hypertonia. The approach suggests that early vertical stimulation and controlled antigravity loading may represent a relevant component of early postural and sensorimotor development. This rationale is theoretically consistent with current evidence on adaptive standing in early intervention, which suggests that upright positioning may contribute to musculoskeletal development, postural symmetry, and activation of antigravity musculature in children with cerebral palsy. When appropriately supported, early standing has also been associated with improvements in hip alignment, bone health, visual exploration, and reduced risk of joint contractures, particularly when combined with active sensorimotor experiences. Such activities may also support sensorimotor integration, especially in children with limited opportunities for spontaneous postural control [50].

From a neuroconstructivist and dynamic systems perspective, delaying upright positioning until certain motor “prerequisites” are met may unnecessarily restrict the child’s interaction with gravitational and task demands. CME instead approaches vertical exposure as a developmental opportunity that may encourage alignment, weight-shifting, and adaptive postural responses in real time. When titrated to the child’s capabilities, this approach may support active engagement with gravity and exploration of functional motor patterns, even in the context of hypertonia or neuromotor delay. This conceptual framework may inform future efforts toward intervention characterization, fidelity assessment, and research reproducibility.

## 3. Toward an Empirical Research Agenda

The conceptual framework proposed in this article supports hypothesis-driven investigation for CME. Importantly, this framework does not provide empirical validation of the intervention, and the proposed mechanisms should be interpreted as hypothetical rather than empirically established pathways.

Current evidence on CME remains limited, consisting primarily of observational studies and clinical reports. The absence of randomized controlled trials, standardized protocols, fidelity measures, and multicenter investigations limits interpretation of current findings. Comparative studies examining CME relative to established pediatric rehabilitation approaches are also currently scarce. In addition, observed improvements may partially reflect non-specific factors such as treatment intensity, therapist expertise, repetition, or caregiver engagement. Clearer operational characterization of CME components is needed, including handling strategies, progression parameters, gravitational challenge calibration, intervention intensity, and intervention fidelity. This may improve reproducibility and comparability across clinical and research settings while preserving the individualized nature of the approach.

The proposed framework may support investigation of hypotheses related to task-related motor activation, anticipatory postural adjustments, multisensory integration, and adaptive motor behavior using complementary biomechanical and neurophysiological measures. Standardized outcomes aligned with contemporary pediatric rehabilitation frameworks should include measures of body function, activity, participation, and caregiver-related outcomes. Potential tools may include the Gross Motor Function Measure (GMFM), Pediatric Evaluation of Disability Inventory Computer Adaptive Test (PEDI-CAT), Canadian Occupational Performance Measure (COPM), Goal Attainment Scaling (GAS), and caregiver-reported measures of participation or quality of life.

Responsiveness to CME may also vary according to biological and developmental constraints, including lesion severity, corticospinal tract integrity, attentional regulation, sensory processing, motivational engagement, and underlying neurological or genetic conditions. These sources of inter-individual variability should be considered when interpreting therapeutic responsiveness and designing future studies. Because CME is a therapist-dependent and individualized intervention, future studies should also consider the influence of therapist training, clinical experience, and implementation variability on treatment fidelity and outcome interpretation.

Safety and tolerability should be systematically monitored during interventions involving progressive verticalization and gravitational challenge. Relevant parameters may include fatigue, pain or musculoskeletal discomfort, emotional distress, excessive compensatory patterns, autonomic signs of intolerance, and the child’s ability to maintain postural organization during task progression. Longitudinal designs with predefined assessment timepoints may further support evaluation of short- and long-term responses to intervention, as well as adjustment of postural and gravitational challenge according to individual tolerance and functional capacity.

Multicenter collaborations across diverse clinical and socioeconomic settings may also support external validity, improve generalizability, and facilitate more equitable access to therapist training and implementation strategies.

## 4. Conclusions

Despite its growing clinical use, CME remains underrepresented in randomized controlled trials and high-level evidence syntheses, reflecting broader challenges in generating and disseminating evidence for complex, therapist-dependent interventions in pediatric neurorehabilitation. Differences in research infrastructure, scientific visibility, and access to resources across contexts may contribute to this gap. As a result, approaches developed in practice-based settings, including those originating in Latin America, may be underrepresented in the literature despite ongoing clinical use. This underscores the need for research strategies that are sensitive to real-world clinical contexts.

In this context, the relationship between therapist training, intervention fidelity, and clinical outcomes should be considered in future research designs, as variability in implementation may influence internal validity and result interpretation. These aspects are particularly relevant for interventions that rely on nuanced clinical reasoning and individualized interaction.

This conceptual perspective does not provide empirical validation, and CME currently lacks robust evidence from randomized trials and standardized protocols. These limitations highlight the need for further investigation to better characterize its proposed mechanisms, therapeutic application, and potential clinical effects using rigorous and context-appropriate methodologies. By aligning CME with contemporary frameworks in motor behavior and developmental neuroscience, this perspective offers a theoretical basis to inform future research, foster interdisciplinary dialogue, and contribute to clinical reasoning in pediatric physical therapy. More broadly, it contributes to ongoing discussions on how practice-based interventions can be conceptually integrated into evidence-informed rehabilitation frameworks. This conceptual reframing may also encourage more transparent clinical reasoning, improve communication across interdisciplinary teams, and support the future development of more structured and reproducible research designs.

## Data Availability

No new data were created or analyzed in this study. Data sharing is not applicable to this article.

## Abbreviations

The following abbreviations are used in this manuscript:

CME	Cuevas Medek Exercise
ICF	International Classification of Functioning, Disability and Health
WHO	World Health Organization
CP	Cerebral Palsy
